# First observation of seasonal variations in the meat and co-products of the snow crab (*Chionoecetes opilio*) in the Barents Sea

**DOI:** 10.1038/s41598-021-85101-z

**Published:** 2021-03-24

**Authors:** Runar Gjerp Solstad, Alexandre Descomps, Sten Ivar Siikavuopio, Rasmus Karstad, Birthe Vang, Ragnhild Dragøy Whitaker

**Affiliations:** 1 NOFIMA-The Norwegian Institute of Food, Fisheries and Aquaculture Research, Muninbakken 9, 9019 Tromsø, Norway; 2 UiT-The Arctic University of Norway, Hansine Hansens vegen 18, 9019 Tromsø, Norway

**Keywords:** Marine biology, Statistics, Biophysical chemistry, Invasive species

## Abstract

The snow crab (*Chionoecetes opilio*), SC, is a newly established species in the Barents Sea. The snow crab fishery has established itself as a new and profitable industry in Norway in the last decade. The fishery started as a year-round fishery, without any information of possible seasonal variations in the quality of the product. In 2017 a total allowable catch was established by the Norwegian government, and the fisheries were subsequently closed during the summer months. In order to optimize fishing times, and to evaluate this growing industry in the Barents Sea, seasonal variations of the meat content of the clusters, as well as variations in content and quality of co-products were investigated, aiming to identify the seasons where the exploitation of different products from SC can be most profitable. The results show seasonal variations in meat content and in composition of co-products. The highest co-product quantities and meat content are from February to April, followed by a period from June to September with decreasing meat and co-products. Our recommendation is to capture the SC in the winter–spring period in the Barents Sea, supporting the current situation and creating most value for the fisheries.

## Introduction

The snow crab (*Chionoecetes opilio*, SC) of the Barents Sea is a new economic resource in Norway. It was discovered for the first time in these waters in 1996 when Russian fishing vessels began reporting SC bycatch^1^. A 2004 survey would later confirm the establishment of the species west of the Russian archipelago of Novaya Zemlya^2^. Commercial fishing for snow crab in the Barents Sea started in 2013. The adaptation of SC as a non-native species in the Barents Sea has prompted the rapid growth of the SC fishery in Norway, with 3.048 metric tonnes landed in 2017 and 4.173 in 2019^3,4^.

The potential of the SC fishery in the Barents Sea depends on the population’s growth and the future spread of the species. Today SC occurs mainly in the eastern part of the Barents Sea, where it inhabits muddy and sand grounds at depths around 200–400 m^2,5^. Since the first observation, SC has gradually spread to inhabit the eastern, central, and north-western Barents Sea^6^. Siikavuopio et al.^7^ concluded that adult male snow crab displays clear behavioural thermoregulation in a hetero-thermal environment, consistently selects temperatures in the coldest end of a thermal gradient (1.0–1.6 °C) and avoid higher temperatures. Consequently, one may expect that snow crab will spread towards the colder north and Svalbard Archipelago as well.

As a relatively new species and commercial resource in the Barents Sea, the biological mapping of seasonal variation in product quality is essential. SC has been a valuable commercial resource in many places, such as the Bering Sea, eastern Canada, and West-Greenland^2^. Only male crabs > 10 cm carapace width are caught. With a high value potential and developed markets in both South Korea and the USA^8^, the biological knowledge on the species can aid commercial fisheries in ensuring a profitable utilisation of this new resource. Additionally, utilising the co-products can lead to added product-value and can benefit the environment and the sustainability of fisheries. The co-products of SC consist of everything except the legs and claws (i.e. clusters): the carapace, cephalothorax, digestive system including the hepatopancreas, and physiological liquid^8^.

This paper aims to present the seasonal variation in meat content, and biochemical composition of the carapace and internal organs defined as the co-products of the SC from five different seasonal points of 1.5 years and provide recommendations to ideal fishing times.

## Methods

### Collection of crabs

Male snow crabs of legal size and with hard shells were caught by the commercial SC vessel Northeastern (Opilio AS) using traditional SC pots in the NEAFC area (N 75° 49.2 E 37° 39.2). SCs were stored live onboard the vessel and subsequently delivered to Nofima’s facilities in Tromsø (N 69° 39). The crabs were caught in June, and September 2016, February, April, and December 2017 and will only be referred to by month. Upon slaughtering, data from individual crabs was obtained by recording the weight of the whole animal, clusters + claws, hemolymph, hepatopancreas, and gills (n = 56 September, n = 45 December, n = 29 February, n = 66 April, n = 50 June). Subsequently, different fractions were pooled and analysed as outlined below.

### Biochemical- and meat content-analyses

The biochemical analyses were performed on each month of analysis (September, December, February, April, and June) and consisted of water, protein, lipid, and ash contents. All analyses were performed on meat (i.e., main product) and the different co-products divided in the following ways: pooled internal organs (mainly hemolymph, hepatopancreas and gonads) with and without added carapace, hemolymph alone and hepatopancreas alone. Lipid class and fatty acid analyses were performed on the lipid storage organ hepatopancreas. Each biochemical analysis consisted of co-products from 10 randomly selected animals. All biochemical analyses (water-, ash-, lipid and protein-content) were determined by Toslab (9266 Tromsø, Norway), lipid classes and fatty acid identifications were performed by Biolab (5141 Fyllingsdalen, Norway). Both are commercial laboratories accredited according to ISO 17025.

#### Water and dry matter content

3–5 g of material was weighed in a marked porcelain crucible. The crucible was placed in a preheated drying cabinet at 103 °C ± 1 °C. After precisely 4 h 30 min, the crucible was allowed to cool down in a desiccator before being weighed. Water and dry matter contents were calculated according to Eqs. (1) and (2) respectively:

1 $$Water\left(\%\right)=\frac{\left(a-b\right)}{w}\times 100$$

2 $$Dry\;matter\;content \left(\%\right)=\frac{\left(b-c\right)}{w}\times 100$$

where a = weight (g) of crucible with weighed sample; b = weight (g) of crucible with dried sample; c = weight (g) of crucible; w = weight (g) of weighed sample^9^.

#### Ash content

3–5 g of material was weighed in a marked porcelain crucible. The crucible was placed in a preheated muffle furnace at 550 °C ± 20 °C. After 16 h, the crucible was allowed to cool down in a desiccator before being weighed. The ash content was calculated according to Eq. (3):

3 $$Ash \left(\%\right)=\frac{\left(d-c\right)}{{w}^{^{\prime}}}\times 100$$

where d = weight (g) of crucible with calcinated sample; c = weight (g) of crucible; w′ =  weight (g) of dry matter sample^10^.

#### Fat content

The fat in the samples was extracted with a polar solvent consisting of CHCl_3_, MeOH and H_2_O in a mixing ratio of 1:2:0.8 to give a single-phase system. 5–20 g of material was weighed into a 250 ml test tube. H_2_O was added so that water content plus added material corresponded to 16 ml. MeOH (40 ml) and CHCl_3_ (20 ml) were added. The mix was homogenized for 60 s. CHCl_3_ (20 ml) was again added and the mix was homogenized for 30 s H_2_O (20 ml) was added, and the mix was homogenized again for 30 s. The test tube was sealed and cooled in a water bath with ice. The emulsion was quickly filtered out through a small cotton ball in a funnel. The upper layer of the collected liquid consisting of MeOH and H_2_O was removed by suction. 5–20 ml of the remaining CHCl_3_ phase was transferred to a tared evaporation dish with a positive displacement pipette. The solvent was evaporated with an infrared lamp. The dish was cooled in a desiccator and weighed. The fat content was calculated according to the Eq. (4):

4 $$Fat\;content \left(\%\right)=\frac{d\times b}{W \times \left(c-\frac{d}{\mathrm{0,92}}\right)}\times 100$$

where b = ml CHCl_3_ added; c = ml CHCl_3_ transferred; d = weight of fat in evaporation dish (g); 0.92 = specific gravity for fat, g/ml; w = weight (g) of the sample^11^.

#### Protein content

Protein content analysis was performed with a fully automated Kjeltec 8400 (Foss Analytics, Denmark). 0.5–1 g of nitrogen free paper of previously dried sample was allowed to be digested in a digestion unit with concentrated H_2_SO_4_ (17.5 ml) and two catalyser tablets for 2 h 20 min at 420 °C. The digested liquid was transferred to the titration unit after cooling and was titrated fully automated by the equipment.

Blanking was performed only with nitrogen free paper, titration with standardized HCl solution and 1% (^w^/_w_) boric acid solution containing a pH sensitive indicator. The protein content was calculated according to the Eq. (5):

5 $$Protein\;content \left(\%\right)=\frac{\mathrm{14,007}\times N \times f \times \left(a-b\right)}{w\times 1000}\times 100$$

where 14.007 = atomic weight of Nitrogen; N = Normality of the titration solution; f = protein factor (6.25); w = weight (g) of weighed sample; a = ml of HCl consumed for sample titration; b = ml of HCl consumed for blank titration^12^.

#### *Cis*-fatty acid and *trans*-fatty acids composition

This method was designed to determine the fatty acid composition of marine oils and marine oil esters in relative (area-%) values, and eicosapentaenoic acid (EPA) and docosahexaenoic acid (DHA) in absolute (g/100 g) values using a bonded polyglycol liquid phase in a flexible fused silica capillary column. C_23:0_ fatty acid was used as an internal standard.

For methyl esterification of oil samples for the analysis of *cis-*fatty acids, two drops of the oil sample were weighed and transferred to a 15 ml test tube with a screw cap. The test amount should be between 20 and 35 mg. Exactly 900 µl of the internal standard solution was added. The solvent was evaporated by nitrogen on a heating block at 80 °C. NaOH solution (1.5 ml, 0.5 N) was added. The mix was incubated in boiling water for 5 min and cooled in cold water. A 15% BF_3_-solution (2 ml) was added. The mix was again incubated in boiling water for 30 min and cooled to 30–40 °C. Isooctane (1 ml) was added. A cork was set, and the mixture grated with gentle movements for 30 s. Saturated NaCl (5 ml) was added immediately. A cork was set, and the mixture was again grated with gentle movements for 30 s. The isooctane phase was transferred to a test tube with a lid. The test tube was centrifuged at 3000 rpm if phase separation was difficult to achieve. Another 1 ml of isooctane was added to the test tube. A cork was set, and the mixture grated with gentle movements for 30 s. The isooctane phase was transferred to the same test tube with a lid. 5 µl of this transferred isooctane phase was diluted into a new test tube with 1 ml of isooctane.

The procedure for methyl esterification of *trans*-fatty acids was identical to that for methyl esterification of *cis*-fatty acids, with one exception: incubation time after the addition of BF_3_-solution was 5 min.

For the GC analysis an analytical capillary column (60 m × 0.25 mm × 0.25 µm-70% Cyanopropyl Polysilphenylene-siloxane) was used. (P/N: 054623, manufacturer: SGE). During the analysis the gas valves on the wall panel for synthetic air and hydrogen were left open.

Two different GC programs were used for the analysis (Table 1).

**Table 1:** GC-program for analysis of fatty acids.

Ramp	Rate (°C/min)	Temp (°C)	*Iso-time* (min)
**For cis-fatty acids**			
Int	–	60	4.00
1	30.0	160	0.00
2	1.0	215	0.00
3	120	260	8.00
**For trans-fatty acids**			
Int	–	60	4.00
1	30.0	160	0.00
2	1.0	215	0.00
3	120	260	8.00

The identification of the different fatty acid methyl esters was performed by comparing the pattern and relative retention times by chromatography of different standards. Empirical response factor was used in quantifying fatty acids, based on calibration solution analysis with equal amounts of included fatty acid methyl esters (GLC-793, Nu-Chek-Prep Inc. Elysian MN, USA). It was calculated according to the Eq. (6):

6 $${RF}_{em }=\frac{{A}_{23:0}}{{A}_{FS}}$$

The absolute amount of each fatty acid, calculated as fatty acid methyl ester was calculated according to the Eq. (7):

7 $${C}_{FS}(g/100)=\left(\frac{{A}_{FS }\times {IS}_{W} \times {RF}_{em} }{ {A}_{23:0} \times W}\right)\times 100$$

where A_FS_ = Area of the fatty acid A_23:0_ = Area of internal standard; IS_W_ = Number of milligrams (mg) internal standard added; RF_em_ = Empirical response factor to the fatty acid with reference to 23:0; W = Weighed sample amount in milligrams (mg); 100 = Factor for conversion to g/100 g^13–15^.

#### Lipid classes

The dominant lipid classes were separated by HPLC equipped with a LiChroCART 125-4, diol 5 µm column and a Charged Aerosol Detector (CAD), using a tertiary gradient mobile phase composition. The fat was extracted as previously described (“Fat content”). A suitable amount of CH_3_Cl was added to the fat sample, the mix was pipetted into a tared test tube and evaporated on a heating block under nitrogen. The temperature of the heating block must be at 60 °C. The test tube with the evaporated sample was weighed and the weight of the fat calculated. The sample was diluted with an appropriate amount of CH_3_Cl. Prior to injection the CAD detector was programmed with these settings: range = 500, Filter = Med, Offset = 5, T = 30 °C. The gradient profile is shown Table 2.

**Table 2:** Gradient profile for separation of lipid classes.

Step	Time (min)	Flow	Canal A	Canal B	Canal C	Canal D	Curve
0	1.0	1.6	100	0	0	0	–
1	7.0	1.6	90	10	0	0	1
2	3.0	1.6	70	30	0	0	1
3	2.0	1.6	40	50	10	0	1
4	13.3	1.6	39	0	61	0	1
5	0.1	2.0	40	0	60	0	1
6	2.0	2.0	40	0	60	0	0
7	2.5	2.0	0	100	0	0	0
8	10.00	2.0	100	0	0	0	0

The quantification was based on external standards with a purity ≥ 98%. Triacylglycerols (TAG) in natural marine oils have a large elution range compared to the other lipid classes, therefore a standard control oil (fish oil) for the preparation of the TAG standard curve was used. This provides a better adaptation to real samples compared to a pure TAG compound.

#### Meat content

Meat content was measured on cooked clusters from the middle of the merus on the first walking leg as an area-percentage of meat-to-shell using an elliptic area formula; Internal height and width of shells and external height and width of muscle was measured, width (w) and height (h) were multiplied to each other and π to calculate the elliptical areas (n = 56 September, n = 45 December, n = 29 February, n = 66 April, n = 50 June). The meat content (MC) was defined as the percentage of space occupied by meat according to the Eq. (8) and the hepatopancreas index (HI) was calculated according to the Eq. (9):

8 $$MC\left(\%\right)=\frac{h\;muscle\times w\;muscle\times \pi }{h\;shell\times w\;shell\times \pi }\times 100$$

9 $$HI\left(\%\right)=\frac{{W}_{hept}}{{W}_{live}}\times 100$$

where W_hept_ is the weight of the hepatopancreas and W_live_ is the live weight of the crab (n = 56 September, n = 50 December, n = 70 February, n = 70 April, n = 50 June).

Graphs, ordinary one-way ANOVA, and linear regressions were made using GraphPad Prism version 7.03 (CA, USA). Meat content data failed the normality test (Shapiro–Wilk) and was analysed using Kruskal–Wallis One Way Analyses of Variance on Ranks and statistical significance was assumed when *P* < 0.05^16,17^.

## Results

### Weight-measurements and analyses

**Figure 1:**
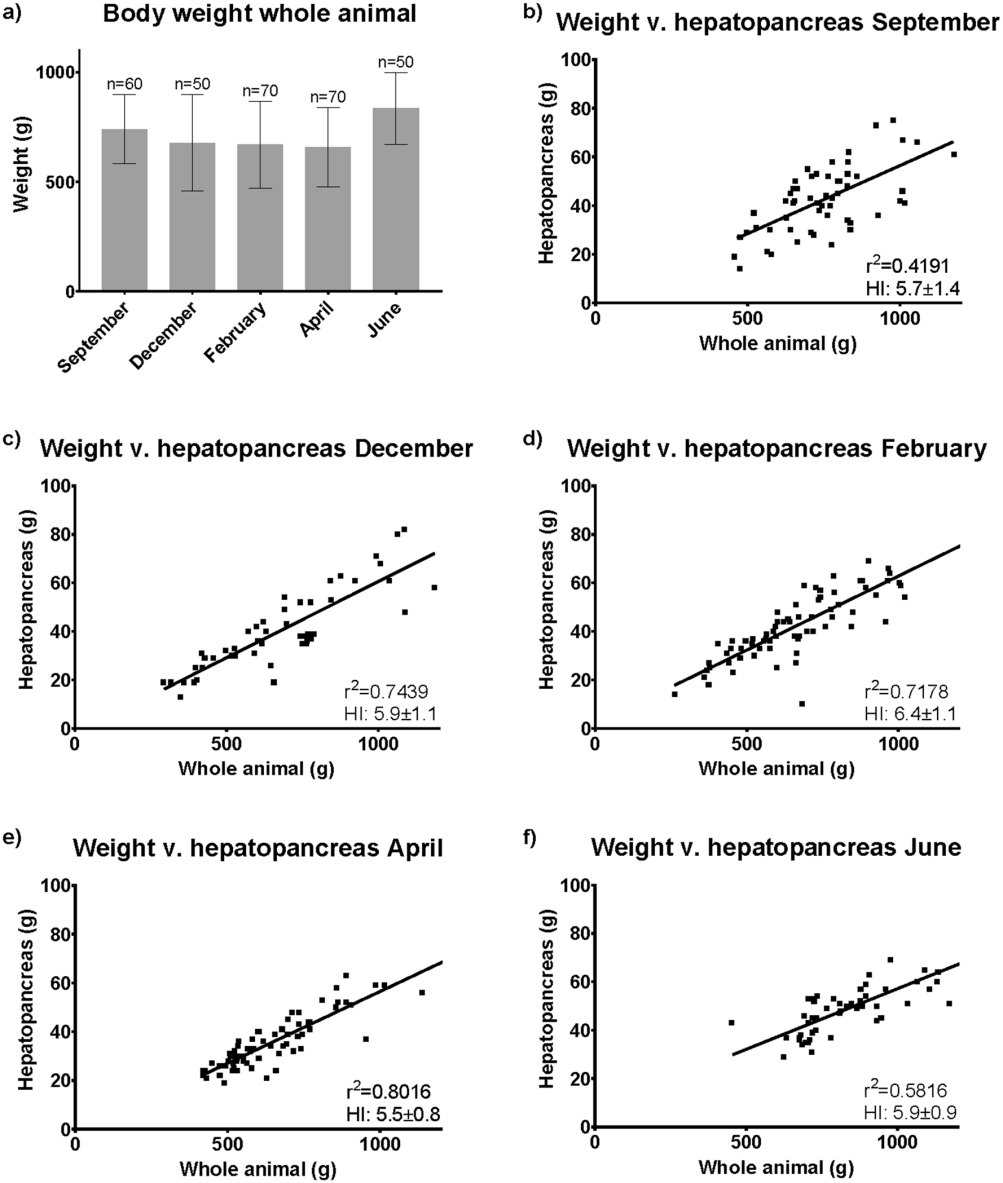
Whole weight and HI. Average weight of whole crab (g) and sample size (n) in all months of sampling (**a**) and comparative analyses of whole-body weight versus hepatopancreas in September, December, February, April, and June (**b**–**f**). Goodness of fit (r^2^) and hepatopancreas index (HI) is also given for each comparison.

The only significant difference in wet weight between samples was seen in June where the animals were significantly larger (P < 0.001, K–W test) compared to other sampling months. The catches are representative of Norwegian commercial fisheries, where the mask width of the pots allows for crabs < 10 cm carapace width to escape. This is illustrated well here as the smallest crab was 260 g (visible in Fig. 1d).

**Figure 2:**
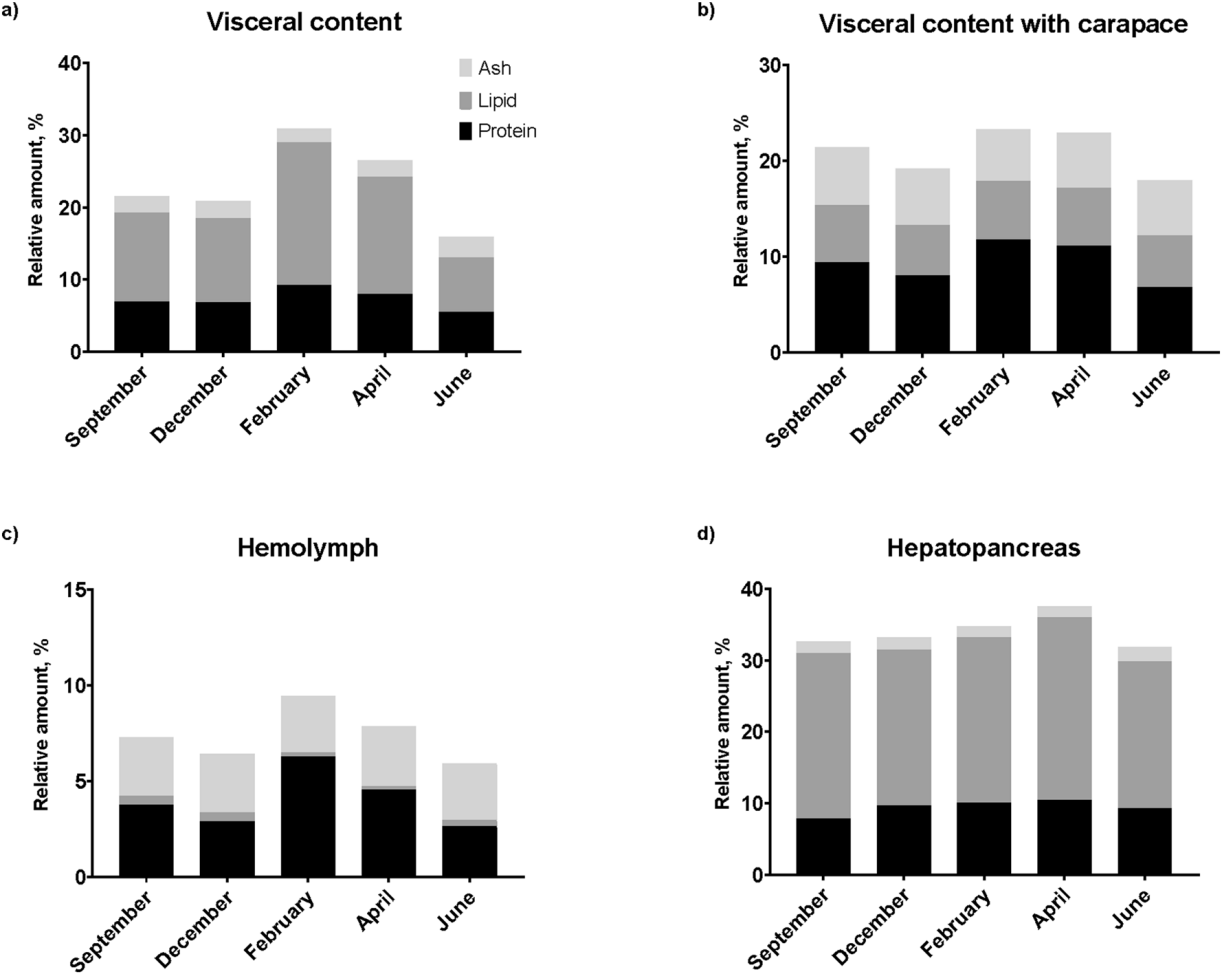
Biochemical parameters of co-products. Measured biochemical parameters of the co-products, i.e., Carapace and internal organs, in all months of sampling. Water, lipid, protein, and ash presented as relative amounts of each in each month of collection. Water is not depicted but is the remaining up to 100. Each bar is represented by 10 pooled and homogenized individuals of: internal organs (**a**), internal organs + carapace (**b**), hemolymph (**c**) and hepatopancreas (**d**).

The hepatopancreas index (HI) was only significantly higher in February (Fig. 1d) compared to April, June, and September (P < 0.001, ANOVA H = 29.699 DF = 4, Fig. 2b,e,f).

### Meat content and biochemical parameters

The MC was measured in clusters during all sampling months (Table 3). There was no statistically significant difference between the February and April groups (K–W test) and these groups displayed significantly higher MC compared to the rest. September and December were quite similar, with median at 83 and 82% respectively and had MC higher than June. June displayed significantly low meat content (K–W test, P < 0.001) compared to the rest. The period from February to June represents the most notable decline.

**Table 3:** Median MC (%) measured in each month of sampling.

Group	n	Median	25%	75%
September	56	0.83	0.77	0.87
December	45	0.82	0.73	0.88
February	29	0.90	0.86	0.95
April	66	0.90	0.84	0.93
June	50	0.78	0.72	0.84

Sample size (n) is also given.

The biochemical variations measured on the cooked muscle of different seasons were smaller than the confidence intervals of the analyses, so no difference between sampling months could be detected. However, representative biochemical data were water 78.5%, protein 19.3%, lipid 0.6% and ash 1.5%.

### Biochemical parameters of co-products

The biochemical parameters measured were water, protein, lipid, and ash. Water makes up the largest part of the crab and its co-products but is not presented here as the discussion largely involves the other biochemical parameters. When comparing the internal organs throughout the different months of sampling (Fig. 2a), the ash content remained stable (1.9–2.9%), whereas protein- and lipid-levels fluctuated more, ranging from 5.5 to 9.3% and 7.5 to 19.7% respectively. Protein- and lipid-levels both peaked in February, where ash- and water-levels were at their lowest (1.9 and 68.1% respectively). The high protein-content in February is a trend that was also recorded in the hemolymph (Fig. 1c), meaning that the hemolymph contributes to this tendency. The abovementioned fluctuating lipid-levels is not equally visible in the samples taken from hepatopancreas alone (Fig. 1d), where the levels are more stable.

With the inclusion of carapaces in the analyses (i.e., internal organs with carapace, Fig. 1b), the image was somewhat different. Lipid- and ash-levels were rather stable throughout the year, whereas protein-levels peaked in February like that of analyses performed on internal organs exclusively.

The hemolymph ash- and lipid-levels (Fig. 1c) were stable at 2.9–3.1% and < 0.5–0.5%. Comparable to other analyses, the hemolymph protein levels fluctuated more and peaked in February, like the other samples. It is unexpected to discover lipids in the hemolymph, and the small quantities present may be due to manual separation of the hepatopancreas and the hemolymph during sampling, making organ cross-contamination very difficult to avoid. Thus, the lipid displayed in the hemolymph (Fig. 1c) is likely due to sampling or analytical error and not the actual amount of lipid in the hemolymph.

All the measured parameters remained consistent in the hepatopancreas throughout all seasons, with June and April representing lowest and highest lipid-levels respectively. The generally higher lipid-levels are expected in this organ due to its storing of energy as lipids.

### Lipid-class and fatty acid composition

The hepatopancreas sample had a high lipid content (Table 4, 21–26%) dominated by more than 80% TAG, small amounts of free fatty acids (FFA) (2–11%), and very little polar lipids (PL 0–5%).

**Table 4:** Seasonal variations in lipid class composition of hepatopancreas (n = 10).

	September	December	February	April	June
Lipid content in sample	23.13 ± 0.52	21.75 ± 0.21	23.14 ± 0.32	25.54 ± 0.31	20.58 ± 0.17
**Lipid classes (g/100 g fat)**					
Triacylglycerol	92.53 ± 0	83.42 ± 2.43	92.31 ± 3.82	93.86 ± 0	81 ± 0
Diacylglycerol	< 0.5	0.87 ± 0.03	< 0.5	< 0.5	< 0.5
Monoacylglycerol	< 0.5	< 0.5	< 0.5	0.02 ± 0.01	< 0.5
Free fatty acids	4.89 ± 0.61	10.52 ± 0.45	3.03 ± 0.84	2.06 ± 0.44	1.5 ± 0
Cholesterol	< 0.5	< 0.5	< 0.5	< 0.5	< 0.5
Cholesterol esters	0.51 ± 0.01	0.52 ± 0.03	< 0.5	0.51 ± 0.01	< 0.5
Phosphatidylethanolamine	< 0.5	< 0.5	0.65 ± 0.05	0.95 ± 0.57	< 0.5
Phosphatidylinositol	< 0.5	< 0.5	< 0.5	< 0.5	< 0.5
Phosphatidylserine	< 0.5	< 0.5	< 0.5	< 0.5	< 0.5
Phosphatidylcholine	< 0.5	< 0.5	3 ± 0.51	3.92 ± 0.62	< 0.5
Lysophosphatidylcholine	< 0.5	< 0.5	< 0.5	< 0.5	< 0.5
Total polar lipids	0 ± 0	0 ± 0	4.1 ± 0.64	4.87 ± 1.19	0 ± 0
Total neutral lipids	97.93 ± 0.62	95.32 ± 2.93	95.34 ± 4.66	96.44 ± 0.45	82.5 ± 0
Total sum lipids	97.93 ± 0.62	95.32 ± 2.93	99.44 ± 5.31	101.3 ± 1.64	82.5 ± 0

The fatty acid analyses were performed on the same samples as the lipid class compositions were determined (Table 5). The fatty acid profiles were quite similar in all hepatopancreas samples without any discernible differences attributed to season. Most of the fatty acids were monounsaturated (35–39%), followed by polyunsaturated (27–28%) and lastly saturated (13–15%). The amount of marine omega-3 fatty acids (EPA and DHA) was at 18–19%.

**Table 5:** Seasonal variation in fatty acid composition of hepatopancreas (n = 10).

Fatty acid (g/100 g)	September	December	February	April	June
*14:0*	2.29 ± 0.01	2.17 ± 0.04	2.29 ± 0.01	2.03 ± 0.02	1.91 ± 0.12
*16:0*	9.61 ± 0.05	9.49 ± 0.15	9.16 ± 0.04	9.47 ± 0.1	11.08 ± 0.57
*18:0*	1.86 ± 0.03	1.91 ± 0.04	1.72 ± 0.01	1.94 ± 0.03	2.1 ± 0.12
*20:0*	0.13 ± 0	0.14 ± 0	0.12 ± 0	0.12 ± 0	0.15 ± 0.01
*22:0*	0.06 ± 0	0.06 ± 0	0.07 ± 0	0.06 ± 0	0.03 ± 0
*16:1 n7*	7.06 ± 0.07	7.12 ± 0.1	5.58 ± 0.02	6.52 ± 0.06	8.11 ± 0.44
*18:1 (n11*+*9*+*7*+*5)*	18.95 ± 0.06	17.16 ± 0.23	17.37 ± 0.04	18.17 ± 0.18	19.63 ± 0.72
*20:1 (n11*+*9*+*7)*	9.74 ± 0.12	9.36 ± 0.02	9.43 ± 0	9.66 ± 0.03	9.04 ± 0.19
*22:1 (n11*+*9*+*7)*	3.3 ± 0.04	2.87 ± 0.01	2.71 ± 0.01	2.36 ± 0	1.5 ± 0.03
*24:1 n9*	0.29 ± 0.01	0.21 ± 0	0.22 ± 0	0.18 ± 0	0.11 ± 0
*16:2 n4*	0.18 ± 0.01	0.21 ± 0	0.12 ± 0	0.15 ± 0.01	0.09 ± 0.01
*16:3 n4*	0.09 ± 0	0.07 ± 0	0.01 ± 0	0.07 ± 0	0.03 ± 0
*18:2 n6*	1.07 ± 0.04	0.73 ± 0.01	0.95 ± 0.02	0.76 ± 0.02	0.51 ± 0.01
*18:3 n6*	0.38 ± 0	0.34 ± 0	0.35 ± 0	0.37 ± 0.01	0.44 ± 0.05
*20:2 n6*	1.29 ± 0	1.16 ± 0.02	1.54 ± 0	1.46 ± 0.01	1.36 ± 0.04
*20:3 n6*	0.16 ± 0	0.15 ± 0	0.15 ± 0	0.16 ± 0.01	0.15 ± 0.01
*20:4 n6*	1.49 ± 0.03	1.57 ± 0.03	1.69 ± 0.01	1.56 ± 0.02	1.32 ± 0.04
*22:4 n6*	0.66 ± 0.01	0.54 ± 0	0.61 ± 0.01	0.6 ± 0	0.67 ± 0.01
*18:3 n3*	0.26 ± 0	0.21 ± 0	0.3 ± 0.01	0.23 ± 0.01	0.3 ± 0.01
*18:4 n3*	0.95 ± 0.02	0.94 ± 0.01	1.05 ± 0.02	1 ± 0.01	0.57 ± 0.03
*20:3 n3*	0.17 ± 0.01	0.13 ± 0	0.16 ± 0	0.13 ± 0	0.15 ± 0
*20:4 n3*	0.43 ± 0	0.42 ± 0	0.49 ± 0	0.44 ± 0	0.4 ± 0
*20:5 n3 (EPA)*	10.85 ± 0.02	12.09 ± 0.17	9.49 ± 0.07	11.23 ± 0.09	11.44 ± 0.27
*21:5 n3*	0.36 ± 0.01	0.39 ± 0	0.37 ± 0.01	0.44 ± 0.01	0.47 ± 0
*22:5 n3*	1.75 ± 0.04	1.63 ± 0.04	1.65 ± 0.01	1.82 ± 0.01	1.9 ± 0.03
*22:6 n3 (DHA)*	7.32 ± 0.04	7.31 ± 0.07	9.12 ± 0.07	7.43 ± 0.05	7.41 ± 0.07
*Sum SFA*	13.96 ± 0.1	13.77 ± 0.23	13.36 ± 0.06	13.62 ± 0.15	15.27 ± 0.8
*Sum MUFA*	39.34 ± 0.29	36.73 ± 0.32	35.32 ± 0.05	36.89 ± 0.27	38.4 ± 1.34
*Sum PUFA (n-6)*	5.06 ± 0	4.5 ± 0.06	5.3 ± 0.04	4.9 ± 0.05	4.45 ± 0.14
*Sum PUFA (n-3)*	22.1 ± 0.13	23.12 ± 0.3	22.64 ± 0.04	22.71 ± 0.17	22.63 ± 0.42
*Sum PUFA total*	27.16 ± 0.14	27.62 ± 0.37	27.93 ± 0.08	27.61 ± 0.22	27.08 ± 0.56
*n-6/n-3*	0.23 ± 0	0.19 ± 0	0.24 ± 0	0.22 ± 0	0.2 ± 0.01
*EPA* + *DHA*	18.17 ± 0.06	19.41 ± 0.24	18.62 ± 0.01	18.66 ± 0.14	18.85 ± 0.33
Sum identified FA	80.72 ± 0.53	78.38 ± 0.91	76.74 ± 0.2	78.34 ± 0.64	80.87 ± 2.71

## Discussion

### Meat content and weight

The MC of June is the lowest observed, the same month when body weight is at its highest and may be related to high energy requirements in preparing for molting, which takes place from July to August in the Barents Sea. Before shredding their confining exoskeleton (premolt), crustaceans stop feeding, and the exoskeletal calcium is solubilized and transferred to the blood and stored in specialized organs. The calcium is later redeposited postmolt into the new exoskeleton to increase its strength^18^. The MC was at its highest in February, which indicates the preferred month for harvesting.

According to Dutil et al.^19^ muscle size and condition of SC are both affected differently from season and year. Condition is said to be mostly affected by season whereas muscle size by year. This may have consequences for the results presented here, especially muscle size (i.e., meat content), since the sampling performed in December falls outside of an uninterrupted 1-year-cycle. Albeit adding uncertainty, it appears that the December sampling aligns to what may be expected in terms of MC, being in the proximity of what was recorded in September and somewhat lower than February.

### Co-products

The co-products of SC consist of several classes of compounds that may be of commercial interest, i.e., Astaxanthin, lipids, proteins or chitin^20^. Biochemical fluctuations were less pronounced in internal organs with carapace included (Fig. 2b) compared to internal organs without carapace (Fig. 2a), which may suggest a biochemical interchange between the internal organs and carapace, perhaps to conserve valuable compounds before molting. Compound interchange has previously been demonstrated in SC with calcium and astaxanthin during proecdysis^21^. The relatively larger ash-levels of internal organs with carapace compared to internal organs alone is very likely a consequence of having the carapace in the analyses as it contains many minerals. In crustaceans, the hepatopancreas is generally regarded as a significant lipid storage organ, and during starvation, body fat, especially from the hepatopancreas, is metabolized^22,23^. Our result demonstrates a change in the hepatopancreas lipid content at different time points. O'Connor and Gilbert^24^ described the variation in depot lipids as a combination of both the external environment and internal conditions. They reported a marked increase in the synthesis and incorporation of lipids in the hepatopancreas in early premolt stages. However, in the late premolt stages, there was a decrease in the lipid content of the hepatopancreas. The lowest lipid content recorded here in hepatopancreas was June (20.3%), increasing during fall and winter months to reach 25.5% in April. The lipid content in the gonads alone was not measured. However, the total lipid content of visceral content will give an overall impression of the total lipid status of the SC. Here we see that again, June has the lowest lipid content with 7.5%, increasing during the fall (September) to 12.3% and winter (December, 11.7%) before reaching a high point in February with 19.7% lipids and declining again to 16.3% in April. According to Harrison^25^, several papers report that during maturation, there is an increase in ovarian lipids due to the mobilization of hepatopancreas lipids. However, there can be several explanations of the depletion of hepatopancreatic lipids, including increased metabolic activity and biosynthetic activity during maturation or a decrease in dietary lipids^25^. There was little difference in HI between seasons (5.5–6.4%, Fig. 1b–f). This indicates that food is not a limiting factor for the growth of SC in the Barents Sea at present^23^.

It can be clearly seen from the results that February is the month where proteins and lipids in the co-products are at its highest levels. Proteins and lipids are the two most common constituent groups to investigate when utilizing the co-products as they are considered to have the greatest commercial value.

### Lipid-analyses

The lipid class analyses are in accordance with other studies that document the lipid classes in crab species^26^. The lipids of the hepatopancreas (Table 4) were stored mainly as TAGs (81–94%) which is common in several crustacean species^27–29^.

Our results show a lipid content of 21–26% (Fig. 2d, Table 4) in the hepatopancreas on a wet weight basis. This is quite high, but corresponding lipid content in hepatopancreas has been found in other crab species^26^. Lipid analyses show few seasonal differences both in the lipid content and in the fatty acid composition (Table 5). The main lipid class in all seasons are TAGs (81–94%) with smaller amounts of other lipid classes present. Latyshev et al.^26^ noted significant fluctuations in the neutral lipid content for the genus *Chionoecetes*, attributing such variations to various factors including access to food, habitation, molting stages, and sex.

## Conclusion

We have evaluated the composition and variations of several biological and chemical parameters from the Barents Sea fisheries of snow crabs landed in Norway, both from meat and co-products. The analyses point to seasonal variations over 1.5 years. The investigations were performed in order to map the differences that can be expected in developing sustainable fisheries of snow crab. In a total-utilisation perspective we also wanted to determine if there were any correlations between optimal meat content, body mass, and co-product compositions, that could indicate whether any seasons are more profitable in the processing of the crabs. Knowing the best season for exploitation of the co-products allows the industry to concentrate efforts around this season to ensure maximum value creation.

The analyses were also performed in order to determine if the crabs are restricted in terms of food access at any time of the year and if the meat content can be correlated to other contents in order to predict the quality of the processed products.

The results presented in this paper point towards the optimal season for catching SC is winter and spring (February–April). The results also indicate that there are no restrictions for food access over the seasons.

The exploitation of co-products is essential to ensure the highest possible degree of sustainability in the fisheries. With this work, we have provided information on the composition of the co-products in different seasons, which may be an aspect to consider in addition to meat quality.
